# Comparative Analysis of Hepatitis C Virus NS5A Dynamics and Localization in Assembly-Deficient Mutants

**DOI:** 10.3390/pathogens10020172

**Published:** 2021-02-04

**Authors:** Laura Riva, Corentin Spriet, Nicolas Barois, Costin-Ioan Popescu, Jean Dubuisson, Yves Rouillé

**Affiliations:** 1 University of Lille, CNRS, Inserm, Institut Pasteur de Lille, CHU Lille, U1019-UMR 8204-CIIL-Center for Infection and Immunity of Lille, 59000 Lille, France; lau.ri.1@tiscali.it (L.R.); nicolas.barois@ibl.cnrs.fr (N.B.); jean.dubuisson@ibl.cnrs.fr (J.D.); 2 University of Lille, CNRS, UMR 8576-UGSF-Department of Functional and Structural Glycobiology, 59000 Lille, France; corentin.spriet@univ-lille.fr; 3 University of Lille, CNRS, Inserm, Institut Pasteur de Lille, CHU Lille, US 41-UMS 2014-PLBS, 59000 Lille, France; 4 Institute of Biochemistry of the Romanian Academy, 060031 Bucharest, Romania; pop@biochim.ro

**Keywords:** hepatitis C virus, NS5A dynamics, NS4B, core, lipid droplets, assembly

## Abstract

The hepatitis C virus (HCV) life cycle is a tightly regulated process, during which structural and non-structural proteins cooperate. However, the interplay between HCV proteins during genomic RNA replication and progeny virion assembly is not completely understood. Here, we studied the dynamics and intracellular localization of non-structural 5A protein (NS5A), which is a protein involved both in genome replication and encapsidation. An NS5A-eGFP (enhanced green fluorescent protein) tagged version of the strain JFH-1-derived wild-type HCV was compared to the corresponding assembly-deficient viruses Δcore, NS5A basic cluster 352–533 mutant (BCM), and serine cluster 451 + 454 + 457 mutant (SC). These analyses highlighted an increase of NS5A motility when the viral protein core was lacking. Although to a lesser extent, NS5A motility was also increased in the BCM virus, which is characterized by a lack of interaction of NS5A with the viral RNA, impairing HCV genome encapsidation. This observation suggests that the more static NS5A population is mainly involved in viral assembly rather than in RNA replication. Finally, NS4B exhibited a reduced co-localization with NS5A and lipid droplets for both Δcore and SC mutants, which is characterized by the absence of interaction of NS5A with core. This observation strongly suggests that NS5A is involved in targeting NS4B to lipid droplets (LDs). In summary, this work contributes to a better understanding of the interplay between HCV proteins during the viral life cycle.

## 1. Introduction

Although efficient direct-acting antivirals were recently approved, hepatitis C remains a major health problem, responsible for nearly 400,000 deaths every year [1]. The causative agent of this disease is the hepatitis C virus (HCV), which is an enveloped positive single-strand RNA virus belonging to the *Flaviviridae* family. HCV genome is about 9600 nucleotides long. It codes for a single polyprotein precursor [2] that is co- and post-translationally processed by both cellular and viral proteases, to generate 10 proteins [3]. Among these, three are structural proteins, which are part of the virion: the capsid protein core and envelope glycoproteins E1 and E2 [4]. The other ones are non-structural (NS) proteins NS2, NS3, NS4A, NS4B, NS5A, and NS5B, and the viroporin p7 [4]. Five of these NS proteins represent the minimal unit required for HCV RNA replication and thus constitute the so-called NS3–NS5B replicase [5], among which NS5B is the RNA-dependent RNA polymerase, which is directly responsible for RNA synthesis [6]. Conversely, NS2 and p7 are mainly involved in HCV assembly, by interacting with both structural and non-structural proteins [7,8].

Similar to other positive-strand RNA viruses, HCV induces membrane rearrangements required for the establishment of a replication compartment called the membranous web (MW), which is characterized by double and multi-membrane vesicles (DMV and MMV) [9]. NS4B is the initiator of the anchoring of the replicase proteins to lipid rafts [10] and is considered to be responsible for the formation of the MW [11]. However, its only expression is not sufficient to induce the formation of DMV [9], suggesting a requirement of the other members of the replicase complex [12]. Then, the newly synthesized RNA is packaged into the nascent virion, whose assembly occurs in proximity of the cytosolic lipid droplets (LDs) [13,14].

NS5A is a multi-functional phospho-protein essential for both HCV genome replication and virion assembly [15]. This protein is localized in the endoplasmic reticulum, in the MW, and on the surface of LDs [14,16]. Its presence at the main sites of both replication and assembly as well as its interaction with the viral RNA [17] and core [18] strongly suggest a role for NS5A as the protein responsible for the transfer of the newly synthetized RNA from the site of its replication to the site of encapsidation [19]. The interaction of NS5A with the core is a crucial step for viral assembly [18] and the core appears to be responsible for NS5A targeting to LDs [14,19,20,21]. NS5A and the core co-localize on the surface of LDs and domain III (DIII) of NS5A is required for this co-localization and for viral assembly [20], while it is dispensable for RNA replication [21]. A recent work also documented the involvement of DI of NS5A in viral assembly and NS5A recruitment to LDs [22]. The basic residues in DI of the core are important for both viral assembly and interaction with NS5A [23,24] and cellular proteins, including the diacylglycerol acyltransferase-1, which also facilitates this interaction [25].

The transition between NS5A functions in genome replication to virion assembly is not clear. In this work, using NS5A-eGFP (enhanced green fluorescent protein) tagged forms of HCV assembly-deficient viruses, we studied NS5A dynamics and sub-cellular co-localization with other viral proteins. We show that NS5A has different motilities and co-localization patterns for viruses blocked in various stages of the virion assembly process. Our work extends the picture of HCV assembly with a dynamic view of NS5A in different stages of the infectious particle formation.

## 2. Results

**Figure 1:**
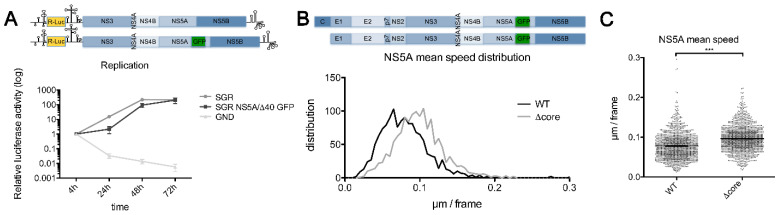
Deletion of core increases NS5A motility. (**A**) Huh-7 cells were electroporated with sub-genomic replicon JFH-1 strain wild type (SGR-JFH-1-WT), SGR-JFH-1/NS5AΔ40/eGFP, and non-replicative SGR-JFH-1-GND, respectively, all expressing *Renilla* luciferase (R-Luc). Samples were harvested at 4, 24, 48, and 72 h post-electroporation and luciferase activity was measured. Error bars represent mean ± SEM for n *=* 3; (**B**,**C**) Huh-7 cells were electroporated with JFH-1/NS5AΔ40/eGFP or JFH-1/Δcore/NS5AΔ40/eGFP and seeded in 15 μ-Slide 8-well chambers. Then, 48 h post-electroporation, cells were video imaged. Results are presented as distribution of the mean speed (**B**) and as a scatter plot (**C**). Error bars represent mean ± SEM for n > 1000 tracks from three independent experiments. A two-tailed Student’s T test was performed as a statistical test (**C**). ***, *p* < 0.001.

In order to analyze the impact of core on NS5A dynamics, we generated a JFH-1-derived sub-genomic replicon, where NS5A was tagged with eGFP. In accordance with published data, aiming to transfer this tag in a wild-type virus, we inserted the compensatory deletion Δ40 in domain II of NS5A, enabling HCV assembly [26]. Thus, we compared the replication level of this replicon to the corresponding untagged one, confirming that the insertion of eGFP in NS5A did not block HCV replication, even if a lag in viral kinetics was observed at early time points (Figure 1A). Therefore, we tagged NS5A in both a JFH-1-derived wild-type virus (JFH-1/NS5AΔ40/eGFP) and a JFH-1-derived virus with a deletion of the protein core (JFH-1/Δcore/NS5AΔ40/eGFP), which are respectively referred to as wild type (WT) and Δcore from now on. Then, the NS5A dynamics were analyzed in parallel in cells infected by these two viruses, using videomicroscopy. As shown in Figure 1B,C, the lack of core strongly increased NS5A motility.

**Figure 2:**
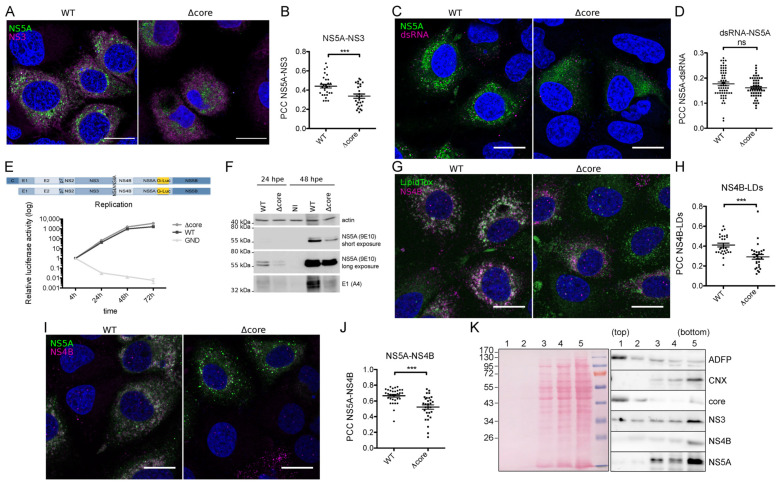
Core increases NS4B association to lipid droplets (LDs) and increases its co-localization with NS5A. (**A**–**D**,**G**–**J**) Huh-7 cells were electroporated with JFH-1/NS5AΔ40/eGFP or JFH-1/Δcore/NS5AΔ40/eGFP and seeded on coverslips. Then, 48 h post-electroporation, cells were fixed and stained with antibodies targeting respectively NS3 (**A**,**B**), double-strand RNA (dsRNA) (**C**,**D**), and NS4B (**G**,**J**). LipidTox was used to stain lipid droplets (LDs) (**G**,**H**). Confocal images of NS5A-GFP and antibody-associated signals were acquired. (**A**,**C**,**G**,**I**). Pearson correlation coefficients (PCC) were calculated on regions of interest (ROIs) from at least 30 different cells in each condition. Scale bars, 20 µm. All the results are presented as means ± SEM. A two-tailed Student’s T test was performed as statistical test (**B**,**D**,**H**,**J**). ***, *p* < 0.001; ns = non-significant. (**E**) Huh-7 cells were electroporated respectively with JFH-1/WT, JFH-1/Δcore/NS5AΔ40, and non-replicative SGR-JFH-1-GND, all expressing *Gaussia* luciferase (G-Luc) inserted in NS5A. Samples were harvested at 4, 24, 48, and 72 h post-electroporation, and luciferase activity was measured. Error bars represent mean ± SEM for n = 3. (**F**) Huh-7 cells were electroporated with JFH-1/WT or JFH-1/Δcore. At 24 h and 48 h post-electroporation, cells were harvested, and a Western blot was performed to analyze the phosphorylation level of NS5A. Actin and E1 (A4 epitope) were respectively used as loading control and infection control. (**K**) Immunoblotting of viral proteins, after purification of LDs of HCV-infected cells by floatation. A Western blot was performed to analyze the core, NS3, NS4B, and NS5A in fractions 1–5 from sampled from the top to the bottom of the gradient. Calnexin (CNX) and ADFP were used as purity controls for endoplasmic reticulum and lipid droplets respectively (right panel). Ponceau staining assessed the total protein level in the five fractions (right panel).

Based on this information, we investigated the reason why the lack of core makes NS5A move faster. As previously reported, the interaction with core influences NS5A recruitment to the lipid droplets (LDs) [14,20,21]. Indeed, HCV deleted of this protein shows a highly reduced presence of NS5A on LDs [14]. Since NS5A is less localized on LDs, we determined whether this protein would be more associated with the replication complexes in the context of Δcore virus. Thus, we immunolabeled NS3, another marker of replication sites [27], and quantified the co-localization of this protein with NS5A. Surprisingly, the virus with its core deleted showed a significant decrease in NS5A–NS3 co-localization (Figure 2A,B). To determine whether NS5A is less associated with the replication complexes in the context of Δcore virus, we immunolabeled the double-strand RNA (dsRNA), an intermediate of HCV RNA replication, and quantified the co-localization between this nucleic acid and NS5A. Very low Pearson’s coefficients were obtained, suggesting that only a small proportion of NS5A labeling overlapped with dsRNA. No statistically significant difference was observed between the wild-type and the Δcore viruses (Figure 2C,D). A replication assay performed with these same viruses where NS5A was tagged with *Gaussia* luciferase instead of eGFP, confirming the absence of difference in replication efficiency (Figure 2E). Therefore, the decreased co-localization between NS5A and NS3 suggests a decrease in the sub-populations of these two proteins that are not involved in RNA replication. In addition, no difference in the phosphorylation level of NS5A was observed (Figure 2F). It worth noting that except if otherwise indicated, the experiments were conducted at 48 h post-electroporation, which is a time point allowing more than one cycle of HCV replication. Since the deletion of core is not compatible with the release of infectious progeny viruses [14], the overall number of replicating cells is expectedly higher in the wild-type compared to the Δcore virus, which is overall reflected by a more abundant accumulation of viral proteins, as documented in Figure 2F. However, we cannot exclude that a reduced electroporation efficiency for the virus lacking core could also be partially responsible for these differences of expression.

NS5A is not the only protein involved in both replication and assembly, which is localized both in endoplasmic reticulum (ER)-derived membranes and in proximity to the LD surface. Indeed, NS4B was described to be involved in the formation of the membranous web [12,28,29], but also to be localized to lipid droplet-associated membranes [30] and influence viral assembly or release [28,31,32]. In order to determine if the lack of core would also affect NS4B in addition to NS5A, we labeled both LDs and NS4B and quantified their co-localization. Similarly to what Miyanari and co-workers reported [14], when the core was absent, NS4B was less present at the surface of LDs (Figure 2G,H). Knowing that NS5A interacts with this NS protein and that this interaction influences NS5A localization to the viral replicase in the endoplasmic reticulum [16], we thus checked if the fact that they were both less recruited to LDs could increase their co-localization level. Surprisingly, a significant reduction in NS5A-NS4B co-localization was observed (Figure 2I,J) in the core-lacking virus, as already observed for NS3.

Immunofuorescence microscopy does not provide the resolution required to assess the localization of proteins directly at the surface of LDs or in membranes that are in close proximity to LDs. To address this issue, we purified by flotation LDs of HCV-infected cells and probed the presence of viral proteins by immunoblotting. As expected, the core was enriched in the top fraction, which also contained the LD marker adipose differentiation related protein (ADFP) (Figure 2K), indicating that most of the core protein was associated with LDs. In contrast, NS4B, NS5A, and, to a lesser extent, NS3 were absent from the LD-enriched fraction and were present in non-floated fractions, that also contained calnexin, an ER membrane marker. This indicated that, conversely to the core, NS proteins that co-localize with LDs in immunofluorescence microscopy are located in membranes that are close to the surface of the LDs, rather than directly bound to the surface of these organelles. Accordingly, the differences of NS proteins co-localization with LDs most likely represent differences of association of NS-containing membranes with these lipid organelles, rather than a change of localization of NS proteins from ER-derived membranes to the surface of LDs.

To better understand the observed phenotypes, we decided to analyze two previously described NS5A-mutant viruses showing a defect during the assembly step: the serine cluster (SC) [20] and the basic cluster (BCM) mutants [19]. In each of these viruses, we inserted an eGFP tag in NS5A, generating JFH-1/NS5AmutBCM-Δ40/eGFP and JFH-1/NS5AmutSC-Δ40/eGFP, respectively called BCM and SC mutant from now on.

In the SC virus, each serine residue of the cluster S452 + S454 + S457 in DIII of NS5A was mutated into alanine, resulting in a defect in NS5A–core interaction and in a lack of recruitment of NS5A to LDs [20]. In the BCM virus, residues from the basic cluster 352–355 in DIII of NS5A were mutated into glutamate, causing an impairment of NS5A-HCV ssRNA interaction and a subsequent defect of ssRNA packaging into the newly formed virions [19].

**Figure 3:**
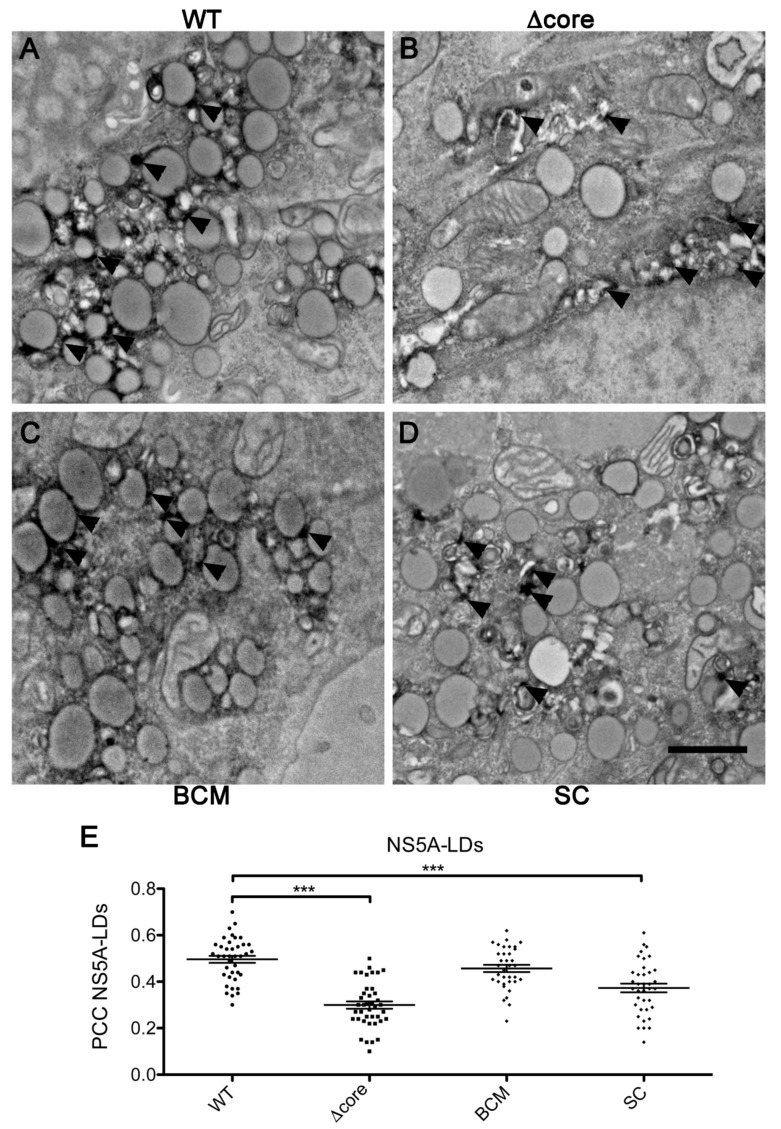
NS5A recruitment to LDs in assembly-deficient viruses. (**A**,**D**) Huh-7 cells were electroporated with JFH-1/NS5AΔ40/APEX2 (**A**), JFH-1/Δcore/NS5AΔ40/APEX2 (**B**), JFH-1/NS5AmutBCΔ40/APEX2 (**C**), and JFH-1/NS5AmutSCΔ40/APEX2 (**D**) and seeded in a 35-mm µ-dish, with an alphanumerical gridded-glass coverslip for the further localization of diaminobenzidine (DAB)-positive cells. Then, 48 h post-electroporation, cells were fixed, incubated with DAB for APEX2 staining, and processed for transmission electron microscopy (TEM). Arrows indicate NS5A localization visualized by APEX2-mediated labeling. Scale bar, 2 µm. (**E**) Huh-7 cells were electroporated with JFH-1/NS5AΔ40/eGFP, JFH-1/Δcore/NS5AΔ40/eGFP, JFH-1/NS5AmutBCMΔ40/eGFP, and JFH-1/NS5AmutSCΔ40/eGFP and seeded on coverslips. At 48 h post-electroporation, cells were fixed and stained with LipidTox and confocal images were acquired. Pearson correlation coefficient (PCC) was calculated on ROIs from at least 30 different cells in each condition. Results are presented as means ± SEM. One-way ANOVA followed by Dunnett post-hoc test was performed as statistical test. ***, *p* < 0.001.

To analyze NS5A intracellular localization of these mutants at a higher resolution, we generated WT and mutant viruses in which the eGFP tag was replaced by APEX2 [33], which is a small peroxidase that allows for a precise labeling of structures containing the NS5A-APEX2 fusion protein in transmission electron microscopy (TEM). The specificity of the APEX2 signal is assessed in Figures S1 and S2. As previously described [34], TEM analyses using an NS5A-APEX-2 labeled virus supported the recruitment of NS5A to LDs (Figure 3A). To note, the surface of the LDs was not uniformly labeled. NS5A was located in dots close to the LD surface that likely represent membrane structures closely associated with LDs, as already suggested by the experiment using LD purification. NS5A in the BCM mutant virus was also positioned close to LDs (Figure 3C), while almost no recruitment was observed for Δcore and SC mutants. In these two latter mutants, NS5A rather displayed accumulation in membranous-derived structures not directly connected to LDs (Figure 3B,D). These observations confirmed the localization of NS5A of WT and BCM viruses at the surface of LDs and the lack of association of NS5A of Δcore and SC viruses with LDs. This was also supported by confocal microscopy analyses using an eGFP-tagged version of all these viruses (Figure 3E).

**Figure 4:**
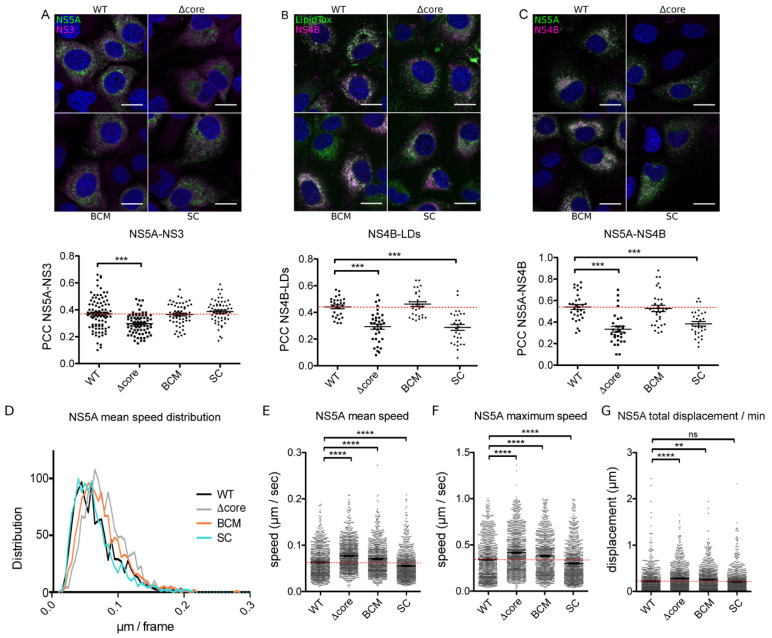
Comparative analyses of NS5A localization and dynamics in assembly-deficient viruses. Huh-7 cells were electroporated with JFH-1/NS5AΔ40/eGFP, JFH-1/Δcore/NS5AΔ40/eGFP, JFH-1/NS5AmutBCMΔ40/eGFP, and JFH-1/NS5AmutSCΔ40/eGFP and seeded on coverslips. At 48 h post-electroporation, cells were fixed and stained with antibodies targeting NS3 (**A**) or NS4B (**B**,**C**). LipidTox was used to stain lipid droplets (LDs) (**B**). Confocal images are shown. Pearson correlation coefficients (PCC) were calculated on ROIs from at least 30 different cells in each condition (**A**,**C** lower panels). Scale bars, 20 µm. All the results are presented as means ± SEM. One-way ANOVA (**A**,**C**) followed by Dunnett post-hoc test was performed as a statistical test. ***, *p* < 0.001. (**D**,**G**) Huh-7 cells were electroporated with JFH-1/NS5AΔ40/eGFP, JFH-1/Δcore/NS5AΔ40/eGFP, JFH-1/NS5AmutBCMΔ40/eGFP, or JFH-1/NS5AmutSCΔ40/eGFP and seeded in μ-Slide 8-well chambers. At 48 h post-electroporation, cells were video-imaged. Results are presented as distribution of the mean speed (**D**) and as scatter plots of the mean speed (**E**), maximal speed (F), and total displacement per minute (**G**). Error bars represent the mean ± SEM for n > 950 tracks from three independent electroporations, and dotted red lines indicate the mean in the wild-type virus. One-way ANOVA followed by Dunnett post-hoc test was performed as statistical test. ****, *p* < 0.0001; **, *p* < 0.01; ns = non-significant.

Using eGFP-tagged versions of BCM and SC mutants, NS5A-NS3 co-localization was also measured and compared to the wild-type and Δcore viruses. No significant difference was observed between BCM or SC mutants and the wild-type virus (Figure 4A). Conversely, looking at NS4B-LDs and NS5A-NS4B co-localization, the SC mutant presented a phenotype similar to the one of Δcore (Figure 4B,C). This supports the hypothesis that NS5A interaction with the core influences not only its recruitment to LDs but also NS4B recruitment. Importantly, these data suggest that the decrease in recruitment of NS4B to LDs in the Δcore virus is not due to the lack of core but rather to NS5A mislocalization, as supported by this same phenotype for the SC virus.

To better determine whether the increase in NS5A motility in the absence of the core was due to the lack of the core protein itself, or to a mislocalization of NS5A and NS4B, or to a more general assembly defect, we also analyzed the dynamics of NS5A in the four viruses in parallel (see supplemental movies). As shown in Figure 4D,E, NS5A moved significantly faster when the virus had its core deleted (mean speed *=* 0.063 ± 0.001 µm/sec in the WT vs. 0.077 µm/sec ± 0.001 in the Δcore virus). In addition, even if to a lesser extent, a significant increase in NS5A motility was also observed with the BCM mutant (0.071 µm/sec ± 0.001). In contrast, a slight reduction of motility was measured for the SC mutant (0.055 µm/sec ± 0.001). These same trends were reflected by the measurement of NS5A maximal speed achieved along the track (Figure 4F). Interestingly, when the total displacement covered by NS5A in one minute was measured, a statistically significant increase was observed in the Δcore (total displacement/min *=* 0.28 µm ± 0.01) and the BCM mutants (0.26 µm ± 0.01), compared to the WT (0.22 µm ± 0.01). Conversely, NS5A SC mutation did not seem to affect this parameter (0.21 µm ± 0.01). These data indicate that the difference of NS5A motility observed with these mutants does not result from differences in the level of interaction between NS5A and the core.

## 3. Discussion

The HCV assembly process represents a complex step of the viral life cycle, which remains incompletely understood. NS5A is a central protein during HCV infection because of its crucial role in both replication and assembly [15]. More specifically, this protein is suspected to be responsible for the switch between RNA replication and encapsidation during HCV assembly [19]. We addressed this issue by studying the dynamics and localization of this protein in assembly-deficient mutants, mainly focusing on the importance of NS5A interaction with core and viral RNA, influencing its intracellular distribution and motility. For this purpose, we used a JFH-1-derived virus containing an eGFP tag in NS5A.

The core is responsible for LD redistribution and influences the dynamics of LDs, decreasing their speed likely in a dynein-dependent manner [35,36]. DII of the core is mainly responsible for this redistribution [37]. NS5A dynamics has also been described to require the microtubules [38] and to be dependent on dynein [39]. In addition, using a trans-complementation system, we recently showed that an exogenous expression of core in a Δcore-eGFP virus alters NS5A dynamics [40]. Here, we report a significant increase in NS5A motility in the absence of a core. The core was reported to directly interact with tubulin and induce microtubule formation in vitro [41]. Therefore, the core could likely impact microtubules function and/or influence the activity of dynein, impacting both LD and NS5A dynamics as a consequence. The study of the NS5A dynamics on the SC mutant supports this hypothesis, documenting motility comparable to the wild-type virus. In the context of this mutation, NS5A is reported to be no longer able to interact with the core and as a consequence to be no longer recruited on LDs. Therefore, it is likely that neither NS5A interaction with the core nor its presence on LDs are responsible for NS5A reduced motility.

Conversely, the observation that BCM also shows a significant increase in NS5A motility, even if to a lesser extent than Δcore, without altering NS5A localization, suggests that the core is not the only determinant impacting NS5A dynamics. Apparently, although the abrogation of NS5A interaction with HCV RNA does not seem to affect NS5A localization, it alters its motility. A previous study described the co-occurrence of large static and small dynamic NS5A-containing structures in replicon-containing cells [38]. The co-localization of HCV RNA and NS5A in both static and dynamic structures has been reported [42]. However, a subset of NS5A motile structures is described to be characterized by the absence of viral RNA [42]. Thus, the increase in NS5A motility in BCM could be due to the increase in the NS5A population not bound to HCV RNA. However, since no alteration in replication was described for BCM [19], this increase in NS5A motility likely suggests that the more static population of NS5A-positive structures could rather be involved in HCV assembly. Indeed, Lee et al. showed that NS5A is recruited around LD in E2-positive punctae, which have decreased mobility comparing to E2-negative NS5A positive punctae [43]. In our previous work, we found also that NS5A peripheral punctae were more mobile than LD proximal NS5A-positive dots [40].

On the other hand, the deletion of the core also led to an alteration of NS5A localization, resulting in a reduction of NS5A recruitment to LDs ([14] and this work) but also to a decrease in NS3–NS5A as well as NS4B-NS5A co-localization (this work). NS3 and NS5A interact and are described markers for the HCV replication compartments [27,44]. In this study, NS5A and dsRNA, an intermediate of replicating RNA, did not significantly co-localize, indicating that a very small proportion of NS5A is present in replication complexes. Therefore, we conclude that the decrease in NS3–NS5A co-localization is not related to actively replicating compartments. It is worth noting that we observed a reduced co-localization of NS3 and NS5A only in the Δcore virus.

NS5A also physically interacts with NS4B [44], and this interaction is important for the proper assembly of the viral replicase and the correct localization of NS5A to this complex [16]. In addition, FRAP (fluorescence recovery after photobleaching) analyses evidenced a role for NS4B in influencing NS5A hyperphosphorylation and motility [28]. Specifically, NS4B deletions in its C-terminus reduce NS5A hyperphosphorylation and increase NS5A fluorescence recovery, while some mutants of this region abrogate the viral replication [28]. In addition, NS4B has been previously described as negatively regulating the NS3–NS5B replication complex [29], interacting with both these proteins [29,45]. In our study, no difference in the viral replication or NS5A phosphorylation is observed upon deletion of the core, although NS4B appears at least partially mislocalized. Conversely, an increase in NS5A motility is observed. However, since the SC mutant shows a level of NS4B relocalization similar to the Δcore mutant but no alteration of NS5A motility, we conclude that the phenotype observed in the Δcore, concerning the dynamics of NS5A, should not be attributed to NS4B.

On the other hand, our data rather suggest an impact of NS5A on NS4B localization and recruitment to LDs. NS4B was in fact described to be also localized on the surface of LDs [14,30] and to have a potential role during viral assembly and/or release. In particular, NS4B was described to bind viral RNA and modulate genome encapsidation [31,46]. In addition, a single-point mutation in the NS4B C-terminus has been described to increase the viral release [28], similarly to mutants of the conserved acidic residues on the hydrophilic side of the N-terminal AH1 helix [32]. Therefore, the reduction in NS4B recruitment to LDs as well as in its co-localization with NS5A in both Δcore and SC mutants strongly suggests that the decrease of NS5A recruitment to LDs, as a consequence of the abrogation of core–NS5A interaction, also impacts NS4B localization, likely indicating an influence of NS5A in targeting NS4B to LDs. Since NS5A and NS4B do not appear to be located directly at the LD surface, but in membrane structures in close proximity to LDs, it is tempting to speculate that they are part of replication complexes. However, quantitative biochemical analyses have also indicated that the majority of HCV non-structural proteins are not incorporated into the HCV replicase [47]. Thus, it is likely that changes in NS proteins’ co-localization are not directly correlated to RNA replication complexes.

In summary, we generated NS5A-eGFP tagged versions of WT HCV and of the three assembly-deficient viruses Δcore, BCM, and SC. Studies of NS5A dynamics highlighted an increase in NS5A motility in the absence of core, which is likely associated with a core-dependent modulation of microtubule and/or dynein-related activity. To a lesser extent, BCM also showed an increase in NS5A speed, which is expectedly due to the abrogation of its interaction with the viral RNA, suggesting an involvement of the less dynamic NS5A population in viral assembly rather than replication. Finally, the reduced co-localization of NS4B with both NS5A and LDs observed in Δcore and SC mutants, which is likely connected to a lack of interaction of NS5A with the capsid protein, strongly suggests a role for NS5A in targeting NS4B to LDs.

Our results support a model where the HCV replication takes place in double-membrane vesicles harboring mobile replication complexes with NS5A, NS4B proteins, and double-stranded RNA. The core protein is inducing profound intracellular membrane rearrangements, positioning the replication and the assembly sites in the proximity of lipid droplets (shown by Lee et al. [43]). The membrane rearrangement is induced by the core–NS5A interaction and the core action on microtubule dynamics. The NS5A-positive replication sites recruited in the LD proximity are involved in HCV particle assembly, and they have a reduced mobility caused by the NS5A-core and NS5A-RNA interactions during the transfer of the viral genome to the nascent viral particle.

Our data bring more insight into the dynamic nature of the HCV assembly process. Further investigations are necessary to characterize the composition of the mobile NS5A positive structures, the molecular mechanisms of their formation, and their role in HCV assembly and replication. Moreover, the molecular switches that drive the NS3, NS4B, and NS5A proteins from their replication to assembly function remain to be elucidated.

## 4. Materials and Methods

### 4.1. Cell Culture

Huh-7 cells have been previously described [48] and were cultured at 37 °C with 5% CO_2_ in Dulbecco’s modified Eagle’s medium (DMEM) supplemented with 10% fetal bovine serum (FBS) and glutamax. Cells were authenticated by the company Multiplexion, performing a Multiplex human cell line Authentication Test (MCA) as described at www.multiplexion.de.

### 4.2. Primers

BCM_R352-355E-F: 5′-CCCCAGAGGAAGAGGAGACAGTGGGTCTGAGCGAGAGCACC-3′

BCM_R352-355E-R: 5′-ACTGTCTCCTCTTCCTCTGGGGGAGGCGTCGGGGCCTTCTT-3′

NsiI-F: 5′-CGCCACATGCATGCAAGCTGAC-3′

XbaI-R: 5′-GTCGACTCTAGACATGATCTGC-3′

APEX2-BglII_F: 5′-AATCAGATCTGGCGGCGACTACAAGGATGACGACGA-3′

APEX2-NruI_R: 5′-AATCTCGCGAGTCCAGGGTCAGGCGCTCCAGGGGA-3′

IRES(+): 5′-CTGTGAGGAACTACTGTCTT-3′

Rluc-PmeI(-): 5′-GAGGGTTTAAACTCATTGTTCATTTTTGAGAACTCGC-3′

### 4.3. Plasmids

All the constructions generated and employed in this study derive from a modified JFH1 plasmid kindly provided by T. Wakita (National Institute of Infectious Diseases, Tokyo, Japan) [49], bearing some titer-enhancing mutations and the reconstitution of the A4 epitope in E1 [50]. The SGR-Rluc replicon was constructed by replacing the sequence of the aminoglycoside 3’-phosphotransferase of pSGR-JFH1 [51] by the sequence of Renilla luciferase amplified by PCR using primers IRES(+) and Rluc-PmeI(-) and inserted in AgeI and PmeI sites. JFH-1/NS5AΔ40/eGFP and JFH-1/Δcore/NS5AΔ40/eGFP were previously described [40]. JFH-1/NS5A-BCM-Δ40/eGFP was generated by two-step PCR-based site-directed mutagenesis using the primers BCM_R352-355E-F, XbaI-R, NsiI-F, and BCM_R352-355E-R, which is followed by enzymatic digestion using NsiI and XbaI. The mutation of serine 452, 454, and 457 of NS5A to alanine residues (JFH-1/NS5A-SC3B) was previously described [8,18]. To construct JFH-1/NS5A-SC-Δ40/eGFP, the Δ40/eGFP coding sequence was inserted in JFH-1/NS5A-SC3B by NsiI/BsrGI enzymatic digestion. JFH-1/WT expressing G-Luc in NS5A was previously described [23]. The corresponding Δcore construction was generated after enzymatic restriction using NsiI and XbaI enzymes’ purification and ligation, respectively, of the NsiI-XbaI fragment from the luciferase construction and the XbaI-NsiI from the Δcore/eGFP one. The same digestion was performed on the JFH-1/NS5AΔ40/eGFP and the SGR-R-luc plasmid to generate the SGR-Rluc-NS5AΔ40/eGFP. For the APEX2-tagged constructions, the APEX2 sequence was amplified from the pcDNA3 APEX2-NES plasmid (Addgene, #49386, Watertown, MA, USA) using primers APEX2-BglII_F and APEX2-NruI_R. Then, the sequence was used to replace the eGFP in the previously described eGFP-tagged viruses, after enzymatic digestion, using BglII and NruI restriction enzymes.

### 4.4. RNA Transcription and Electroporation

After linearization of the plasmids described in the former paragraph using XbaI and treatment with the Mung Bean Nuclease (New England Biolabs, Ipswich, MA, USA) as previously described [8], RNA was in vitro transcribed using MEGAscript^®^ T7 transcription kit (Life Technologies, Carlsbad, CA, USA). Huh-7 cells were electroporated using 10 μg of RNA according to the method previously described [51], using 0.2 cm gap electroporation cuvettes (Biorad, Hercules, CA, USA).

### 4.5. Antibodies and Reagents

Mouse monoclonal antibody (mAb) A4 against HCV E1 was produced in vitro using a MiniPerm apparatus (Heraeus, Hanau, Germany) following the manufacturer’s protocol. Mouse anti-NS3 mAb (486D39) was gently provided by J.-F. Delagneau (Bio-Rad). Anti-NS4B antibody was a kind gift of D. Moradpour and J. Gouttenoire. Mouse anti-dsRNA mAb J2 was purchased from Scicons (Budapest, Hungary). Sheep anti-NS5A antiserum [52] was kindly provided by M. Harris. Anti-sheep-HRP, anti-mouse-HRP, and anti-mouse Cy3 antibodies were purchased from Jackson Immuno-Research. LipidTox Deep Red was obtained from Life Technologies. 4′,6-Diamidine-2′-phenylindole (DAPI) was purchased from Sigma Aldrich (St. Louis, MO, USA).

### 4.6. Videomicroscopy

Huh-7 cells were electroporated with the indicated viral RNAs and seeded in 15-μ-Slide 8-well chambers (Ibidi, Fitchburg, WI, USA). At 48 h post-electroporation, cells were analyzed using the AxioObserver Z1 Video-DG4 Microscope (Zeiss, Oberkochen, Germany). Images were acquired with a 63x oil-immersion objective. Acquisition of each field was performed at one image every 1.2 s during 300 frames. Movies were analyzed using the Fiji plugin TrackMate [53,54]. The Laplacian of Gaussian (LoG) detector was used to identify single spots, setting a cut off in size at 1 µm. Additional filters were set on spots on the bases of local maximum intensity and quality, removing all the weak-intensity particles unsuitable to be unequivocally identified from one frame to the other. Settings were fixed for Simple LAP (Linear Assignment Problem) tracker as follows: Linking max distance *=* 1 µm, Gap-closing max distance *=* 1 µm, Gap-closing max frame gap *=* 5. Tracks shorter than 100 frames were discarded during the analysis. Then, track duration and total displacement, and speed mean, max, mean, and standard deviation were extracted, and the displacement/min was calculated from >950 tracks. Data representation and statistical analysis were performed using GraphPad Prism.

### 4.7. Immunofluorescence

Electroporated Huh-7 cells were seeded on coverslips. After 48 h, cells were fixed for 30 min with 3% paraformaldehyde. Cells were permeabilized during 5 min with 0.1% Triton X-100 and blocked for 30 min with phosphate buffered saline (PBS) containing 10% goat serum. Primary antibodies were incubated for 30 min in PBS–goat serum. After three washes, secondary antibodies and DAPI were added during 30 min. After three more washes, coverslips were mounted on glass slides using Mowiol-containing medium. Images were taken using a Zeiss-LSM880 or a Zeiss-LSM780 confocal microscope with a 63X oil objective. Pearson correlation coefficients were calculated using the Fiji coloc2 plugin on regions of interest (ROIs) from single cells, which were designed on the basis of NS5A or NS4B labeling. Each experiment was repeated three times, and at least 30 cells for each condition were quantified.

### 4.8. APEX2 Labeling and Transmission Electron Microscopy (TEM)

Huh-7 cells were electroporated with the indicated viral RNAs and seeded on a 35-mm µ-dish, with an alphanumerical gridded-glass coverslip (ref P35G-1.5-14-CGRD, MatTek Corporation, Ashland, MA, USA). At 48 h post-electroporation, cells were fixed with 4% paraformaldehyde, 1% glutaraldehyde, and 4% sucrose in 1X PBS for 30 min. Before diaminobenzidine (DAB) reaction, cells were treated with 100 mM glycine in 1X PBS for 20 min. The DAB reaction was done in the dark for 15 min with the Sigma Fast DABTM kit (D4418, Sigma, St. Louis, MO, USA). The reaction was stopped by 1X PBS washes. Before TEM preparation, DAB-positive cells were localized on the gridded coverslip by optical microscopy. Cells were contrasted with 1% osmium tetroxide reduced with 1.5% potassium hexacyanoferrate (III) for 1.5 h followed by 1% uranyl acetate for 1 h, both in water and in the dark. After contrasting, cells were dehydrated in graded ethanol solutions, infiltrated with epoxy resin, and cured at 60 °C for 48 h. After separation of the resin from the glass, cells of interest were relocated with the imprinted-alphanumerical grid at the surface of the resin. Small blocks of resin containing the cells of interest were prepared for sectioning parallel to the resin surface. Serial sections 80-nm thick were set down on carbon/formvar-coated slot grids. Sections were observed with a Hitachi H7500 TEM (Milexia, Verrières-le-buisson, France), and images were acquired with a 1 Mpixel digital camera from AMT (Milexia).

### 4.9. Purification of Lipid Droplets

Huh-7 cells were infected with HCV at a multiplicity of infection (M.O.I.) ≈1 and used 2 days later to purify lipid droplets using the lipid droplet isolation kit from Abcam (ab242290), as indicated by the manufacturer, except that the gradients were centrifuged for 60 min at 100,000× *g* instead of 3 h at 20,000× *g*. Five fractions of 270 µL were sequentially removed from the top of the gradient and stored at −80 °C until Western blot analysis.

### 4.10. Quantification and Statistical Analyses

Data are presented as means ± standard error of the mean (SEM), except when specified differently. Statistical parameters and analyses are reported in the figure legends. n represents the number of independent experiments, except when differently indicated. Statistical analyses were performed using GraphPad Prism 5 and 7.

## Data Availability

The data presented in this study are available on request from the corresponding author.

## Supplementary Materials

The following are available online at https://www.mdpi.com/2076-0817/10/2/172/s1, Figure S1: optical control of APEX expression, after addition of diaminobenzidine to Huh-7 cells electroporated with the indicated viruses (48 h.p.e.). Figure S2: Negative controls for transmission electron microscopy (TEM) evaluating the specificity of NS5A recruitment to LDs in infected cells. Huh-7 cells were electroporated with the indicated virus or mock-electroporated. 48 h later, cells were fixed and diaminobenzidine (DAB) was added to stain APEX-positive cells. Stars indicate lipid droplets. Video SM1: NS5A subcellular tracking in Huh-7 cells electroporated with the WT virus. Video SM2: NS5A subcellular tracking in Huh-7 cells electroporated with the Δcore virus. Video SM3: NS5A subcellular tracking in Huh-7 cells electroporated with the SC virus. Video SM4: NS5A subcellular tracking in Huh-7 cells electroporated with the BCM virus.
