# Supplementary material for: Comparative Analysis of Hepatitis C Virus NS5A Dynamics and Localization in Assembly-Deficient Mutants
Source: Pathogens. 2021 Feb 4;10(2):172. doi: 10.3390/pathogens10020172 (PMC7919264; doi:10.3390/pathogens10020172)
Supplement: Supplementary file 1 [file pathogens-10-00172-s001.zip › Supplementary caption.pdf]

Supplementary caption

Figure S1. NS5A recruitment to LDs in assembly-deficient viruses (light microscopy). (A-D) Huh-7 cells were electroporated with JFH-1/NS5AΔ40/APEX2 (WT-APEX), JFH-1/NS5AΔ40/eGFP (WT-GFP), JFH-1/Δcore/NS5AΔ40/APEX2 (Δcore-APEX), JFH-1/NS5AmutBCΔ40/APEX2 (BCM-APEX) and JFH-1/NS5AmutSCΔ40/APEX2 (SC-APEX) or left untransfected (NI). 48 h post-electroporation cells were fixed, incubated or not with DAB (+DAB and no DAB respectively) for APEX2 staining and imaged using an optical microscope.

Figure S2. NS5A recruitment to LDs in assembly-deficient viruses (electron microscopy). (A-D) Huh-7 cells were electroporated with JFH-1/NS5AΔ40/APEX2 (WT-APEX), JFH-1/NS5AΔ40/eGFP (WT-GFP) or left untranfected (NI). 48 h post-electroporation cells were fixed, incubated or not with DAB (+DAB and no DAB respectively) for APEX2 staining and processed for transmission electron microscopy (TEM). * indicate lipid droplets. Scale bar, 2 µm.
