# Supplementary figures and images for: Comparative Analysis of Hepatitis C Virus NS5A Dynamics and Localization in Assembly-Deficient Mutants
Source: Pathogens. 2021 Feb 4;10(2):172. doi: 10.3390/pathogens10020172 (PMC7919264; doi:10.3390/pathogens10020172)

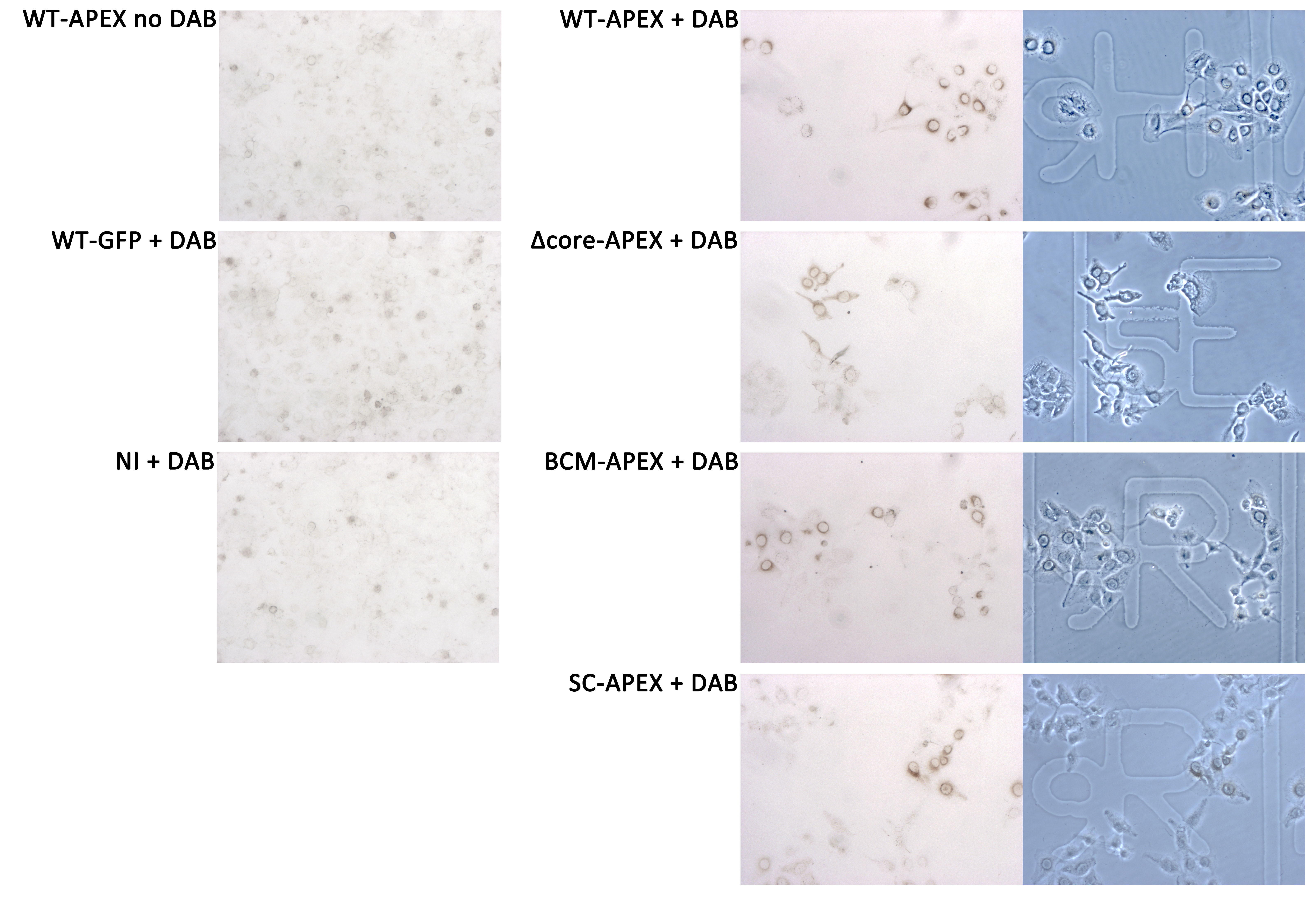

Supplement: Supplementary file 1 [file pathogens-10-00172-s001.zip › Figure S1_Figure.tif]

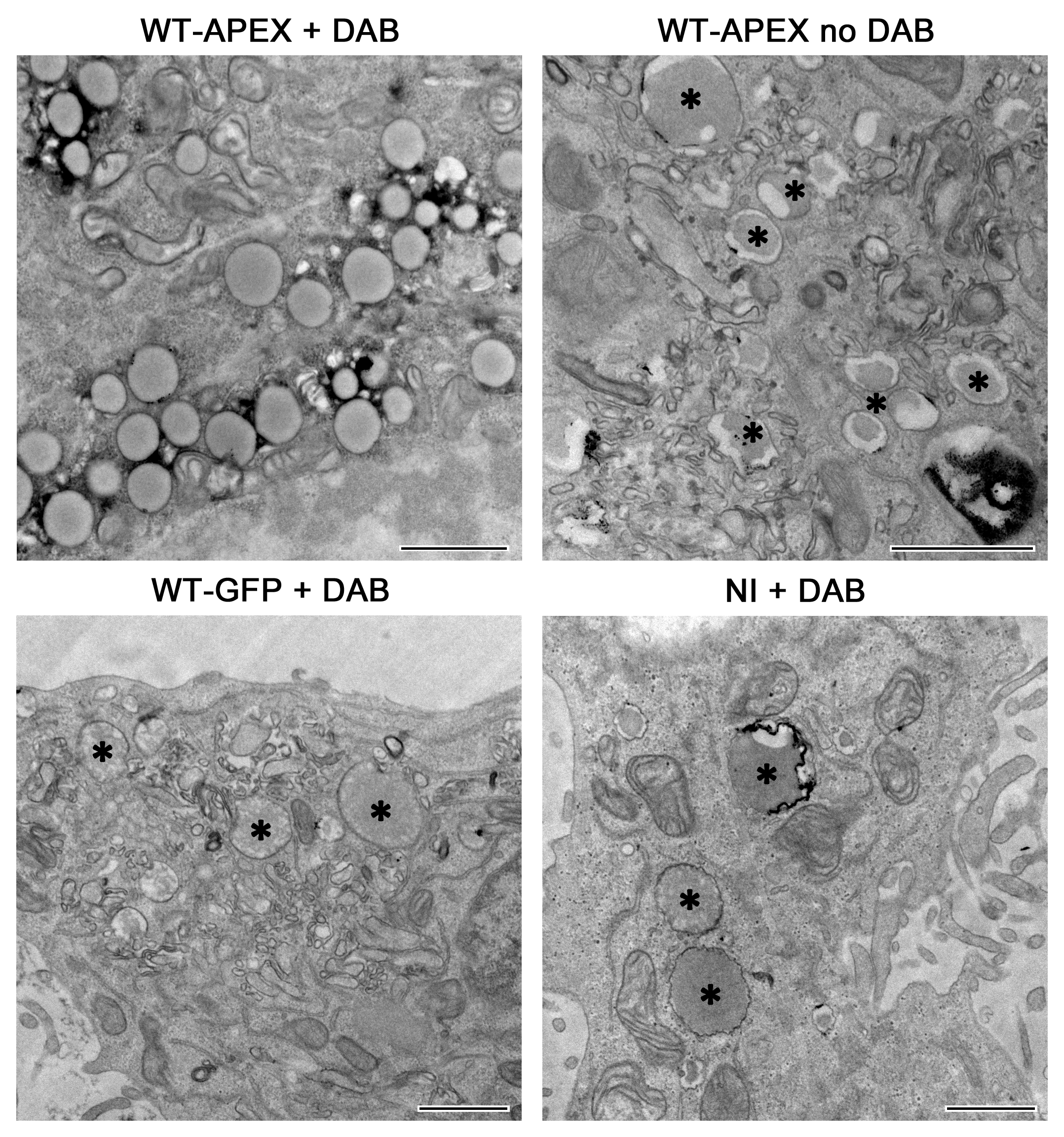

Supplement: Supplementary file 1 [file pathogens-10-00172-s001.zip › Figure S2_Figure.tif]
